# Analyses of the Impact of Immunosuppressive Cytokines on Porcine Macrophage Responses and Susceptibility to Infection to African Swine Fever Viruses

**DOI:** 10.3390/pathogens11020166

**Published:** 2022-01-27

**Authors:** Giulia Franzoni, Susanna Zinellu, Tania Carta, Chiara Grazia De Ciucis, Floriana Fruscione, Antonio Anfossi, Mauro Ledda, Simon P. Graham, Silvia Dei Giudici, Elisabetta Razzuoli, Annalisa Oggiano

**Affiliations:** 1 Department of Animal Health, Istituto Zooprofilattico Sperimentale Della Sardegna, 07100 Sassari, Italy; Susanna.Zinellu@izs-sardegna.it (S.Z.); cartania_75@yahoo.it (T.C.); Silvia.DeiGiudici@izs-sardegna.it (S.D.G.); annalisa.oggiano@izs-sardegna.it (A.O.); 2 Department of Veterinary Medicine, University of Sassari, 07100 Sassari, Italy; aanfossi@uniss.it (A.A.); vetleddamauro@gmail.com (M.L.); 3 National Reference Center of Veterinary and Comparative Oncology (CEROVEC), Istituto Zooprofilattico Sperimentale del Piemonte, Liguria e Valle d’Aosta, Piazza Borgo Pila 39/24, 16129 Genoa, Italy; chiaragrazia.deciucis@izsto.it (C.G.D.C.); floriana.fruscione@izsto.it (F.F.); elisabetta.razzuoli@izsto.it (E.R.); 4 The Pirbright Institute, Ash Road, Pirbright, Woking GU24 ONF, UK; simon.graham@pirbright.ac.uk

**Keywords:** porcine macrophages, African swine fever virus, IL-10, TGF-β, cytokines, flow cytometry

## Abstract

African swine fever viruses (ASFV), currently a serious threat to the global pig industry, primarily target porcine macrophages. Macrophages are characterized by their remarkable plasticity, being able to modify their phenotype and functions in response to diverse stimuli. Since IL-10 and TGF-β polarize macrophages toward an anti-inflammatory phenotype, we analyzed their impact on porcine monocyte-derived macrophages’ (moMΦ) susceptibility to infection and their responses to two genotype I ASFV strains, virulent 26544/OG10 and attenuated NH/P68. At a low multiplicity of infection (MOI), NH/P68, but not 26544/OG10, presented a higher ability to infect moM(IL-10) compared to moMΦ and moM(TGF-β), but no differences were appreciated at a higher MOI. Both strains replicated efficiently in all moMΦ subsets, with no differences at later times post-infection. Both strains downregulated CD14 and CD16 expression on moMΦ, irrespective of the activation status. ASFV’s modulation of CD163 and MHC II DR expression and cytokine responses to NH/P68 or 26544/OG10 ASFV were not affected by either IL-10 or TGF-β pre-treatment. Our results revealed little impact of these anti-inflammatory cytokines on moMΦ interaction with ASFV, which likely reflects the ability of the virus to effectively modulate macrophage responses.

## 1. Introduction

African swine fever (ASF) is a viral disease of domestic pigs and wild boar that threatens the global pig industry due to the lack of treatment or vaccines, with ongoing outbreaks in Africa, Europe, Asia, Oceania, and most recently in the Americas [1,2]. The aetiological agent is the African swine fever virus (ASFV), a large, double-stranded DNA virus (170–190 kb), which is the only known DNA arbovirus and the sole member of the *Asfarviridae* family [1]. 

Monocytes and macrophages (Mφ) are the main target cells for ASFV [3]. Macrophages are a key element of the innate immune system, and virulent ASFV isolates have evolved mechanisms to modulate macrophage responses, allowing them to survive and effectively replicate in these cells [3]. On the contrary, macrophage infection with attenuated ASFV strains leads to the increased expression of key cytokines and chemokines, which could trigger the induction of innate immune responses and promote the development of effective adaptive immune responses [3,4]. 

Mφ are characterized by their remarkable plasticity and are able to quickly modify their phenotype and functions in response to the changes in the surrounding environment [5]. The two extremes of their diverse polarized states are represented by ‘classically’ (M1) and ‘alternatively’ (M2) activated Mφ, which can be generated in vitro using IFN-γ and LPS, or IL-4, respectively. M1 are characterized by antimicrobial and tumoricidal activities, whereas M2 are mainly associated with mechanisms of immunosuppression and wound healing [5]. M2 macrophages were later divided into different subsets (M2a, M2b, and M2c) [6], but more recently, further differences were observed between the polarizing effects of the different stimuli; thus, the scientific community was encouraged to adopt a nomenclature linked to the activator(s) used, e.g., M(IL-4), M(IFN-γ), M(IL-10), M(LPS), M(Ig) [7].

To date, few studies have investigated the interaction of ASFV with macrophages in distinct activation states. It was recently reported that macrophage polarization with IL-4 did not affect their responses and susceptibility to ASFV infection, whereas M1 activation (with IFN-γ and LPS) of either porcine alveolar macrophages (PAM) or monocyte-derived macrophages (moMφ) resulted in delayed ASFV replication [8,9]. 

IL-10, TGF-β, or glucocorticoids polarize macrophages toward an immunosuppressive phenotype, with the downregulation of pro-inflammatory cytokines and increased debris scavenging activity [10]. We recently described how the treatment of porcine moMφ with IL-10 and TGF-β induced an immunosuppressive phenotype, which was able to trigger MHC II DR and CD14 downregulation and reductions in IL-6 and TNF-α gene expression [11]. Thus, in this study, we analyzed the impact of these immunosuppressive cytokines on macrophage susceptibility and responses to infection with attenuated or virulent ASFV strains. In addition, a high level of IL-10 was detected in the serum of domestic pigs and wild boar infected with virulent ASFV isolates, especially in the final stage before death [12,13,14], suggesting a role for this cytokine in ASFV pathogenesis. 

In this study, we compared the responses of porcine moM(IL-10) and moM(TGF-β) to the highly virulent Sardinian isolate 26544/OG10 and the attenuated NH/P68. The former, isolated in 2010 from naturally infected pigs during ASF outbreaks in Sardinia (in the Nuoro province), induced death in 10–14 days when administered to domestic pigs at very low doses (10 TCID_50_ using an intramuscular inoculation route) (De Mia, unpublished results), whereas infection with the latter provided pigs with protection against subsequent homologous and heterologous ASFV virulent challenge [1,15]. NH/P68 is regarded as a valid tool to understand the induction of protective immune responses against ASFV [1,16]. The ability of these two ASFV strains to infect, replicate, and modify the phenotype and functionality of macrophages pre-treated with immunosuppressive cytokines was assessed through a combination of multiparametric flow cytometry, virus titration, and multiplex and conventional ELISA.

## 2. Results

### 2.1. Impact of IL-10 and TGF-β on Porcine Macrophage Innate Immune Gene Expression

**Figure 1:**
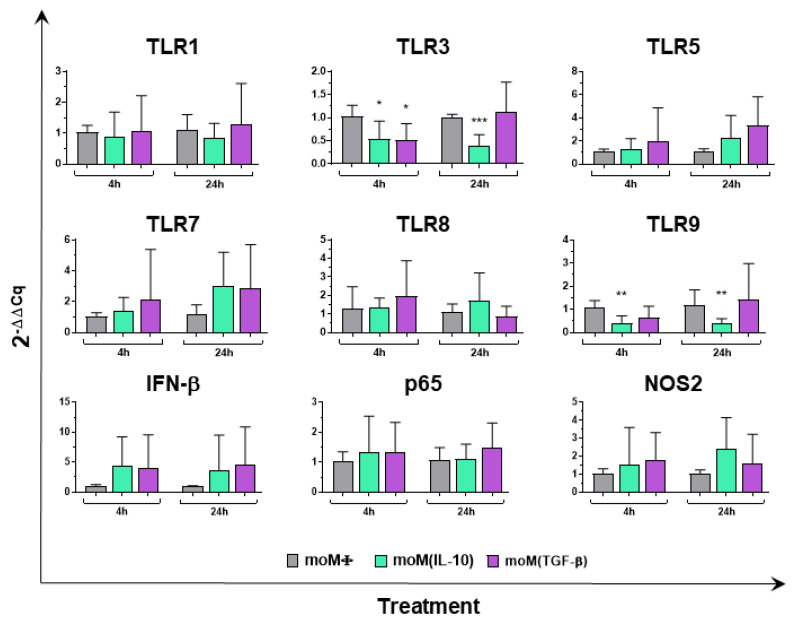
IL-10 and TGF-β impact on porcine macrophage expression of key innate immunity genes. moMΦ were stimulated with recombinant porcine IL-10 or TGF-β, alongside untreated control moMΦ. At 4 and 24 h post-stimulation, RT-qPCR was employed to determine gene expression levels of *TLR-1, TLR-3, TLR-5, TLR-7, TLR-8, TLR-9, IFN-β, p65, NOS2*. At each time point, data were normalized on the values of untreated control (moMΦ) and expressed as 2^−ΔΔCq^, with ΔCq = Cq (target gene)—Cq (reference gene), and ΔΔCq = ΔCq (stimulated samples)—ΔCq (untreated sample, moMΦ). Mean data and SD from five independent experiments using different blood donor pigs are shown. For both 4 and 24 h post-stimulation, values of IL-10 or TGF-β stimulated samples were compared to the corresponding untreated control (moMΦ) using a Kruskal–Wallis multiple comparison test. *** *p* < 0.001, ** *p* < 0.01, * *p* < 0.05.

MoMΦ were generated from peripheral blood monocytes and were left untreated (moMΦ) or were polarized with IL-10 or TGF-β. Phase contrast microscopy and May–Grunwald–Giemsa staining revealed that both the immunosuppressive cytokines did not alter the morphology of these cells (Figure S1a,b), supporting the results of our recent study [11]. In addition, both IL-10 and TGF-β did not alter moMΦ viability (Figure S1c). In the first part of this work, the effects of these immunosuppressive cytokines on porcine moMΦ were further investigated. The gene expression of selected Toll-like receptors (TLRs), IFN-β, p65 (a subunit of transcription factor NF-κB involved in TLR signaling [17]), nitric oxide synthase 2 (NOS2, which encodes for the enzyme that generates nitric oxide (NO) from arginine [18]) were analyzed at 4 and 24 h post-simulation. RT-qPCR data showed that neither IL-10 nor TGF-β induced an enhanced expression of any of the tested genes. On the contrary, stimulation with either IL-10 or TGF-β decreased *TLR3* expression 4 h post-stimulation. In addition, stimulation with recombinant IL-10, but not TGF-β, resulted in the statistically significant downregulation of *TLR3* (24 h post-stimulation) and *TRL9* (4 and 24 h post-stimulation) gene expression (Figure 1). 

### 2.2. Impact of IL-10 and TGF-β on moMΦ Susceptibility to ASFV Infection

**Figure 2:**
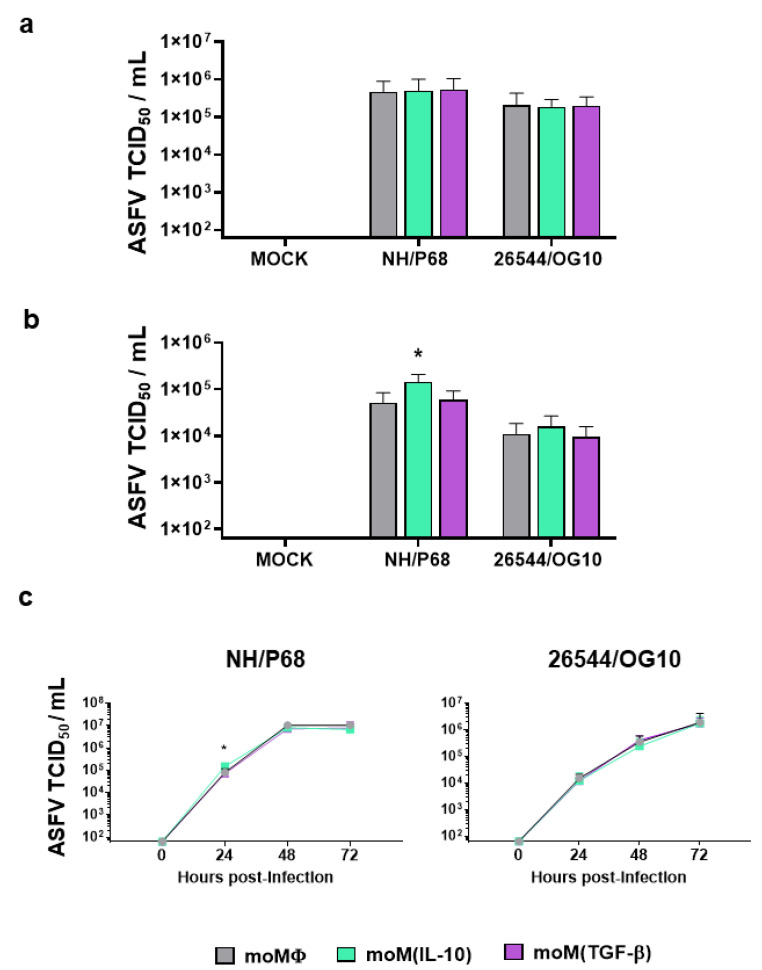
Susceptibility to infection and growth kinetics of ASFV strains of diverse virulence in macrophage subsets. moMΦ, moM(IL-10), moM(TGF-β) were mock infected or infected with the attenuated NH/P68 or the virulent 22544/OG10 ASFV strains using an MOI of 1 (**a**) or 0.01 (**b**,**c**). At 21 hpi (**a**,**b**) or at 0, 24, 48, 72 hpi (**c**), culture supernatants were collected, and the levels of infectious viral progeny were determined by titration (TCID_50_/mL). The mean data + SD from three independent experiments utilizing different blood donors are shown. At each time point and for each isolate (NH/P68 or 26544/OG10), values of treated macrophages were compared to the corresponding untreated control (moMΦ) using a one-way ANOVA followed by Dunnett’s multiple comparison test. * *p* < 0.05.

We then moved to assess the impact of these two immunosuppressive cytokines on macrophage interactions with ASFV. First, the impact of IL-10 and TGF-β on macrophage susceptibility to ASFV infection was assessed by the quantification of viral titers in cell culture supernatants (Figure 2). Macrophage subsets were mock infected or infected with the attenuated NH/P68 or the virulent 26544/OG10 ASFV, using a multiplicity of infection (MOI) of 1 or 0.01. Then, 21 hpi, the amount of infectious virus present in culture supernatant, was determined by titration. As displayed in Figure 2a, for both isolates, no statistically significant differences were detected between macrophage subsets using an MOI of 1. However, at the lower MOI (0.01), slightly higher NH/P68 titers were observed in moM(IL-10) compared to the other subsets (Figure 2b).

We next conducted a kinetic analysis of the infection with either NH/P68 or 26544/OG10 ASFV in moM𝜑, moM(IL-10), moM(TGF-β) using an MOI of 0.01. Both isolates replicated efficiently in all macrophage subsets, regardless of the pretreatment with immunosuppressive cytokines. IL-10 stimulation resulted in increased levels of virus supernatants in NH/P68-infected moMΦ at 24 hpi, whereas for both strains, no differences were appreciated between subsets at later time points (48 and 72 hpi) (Figure 2c).

**Figure 3:**
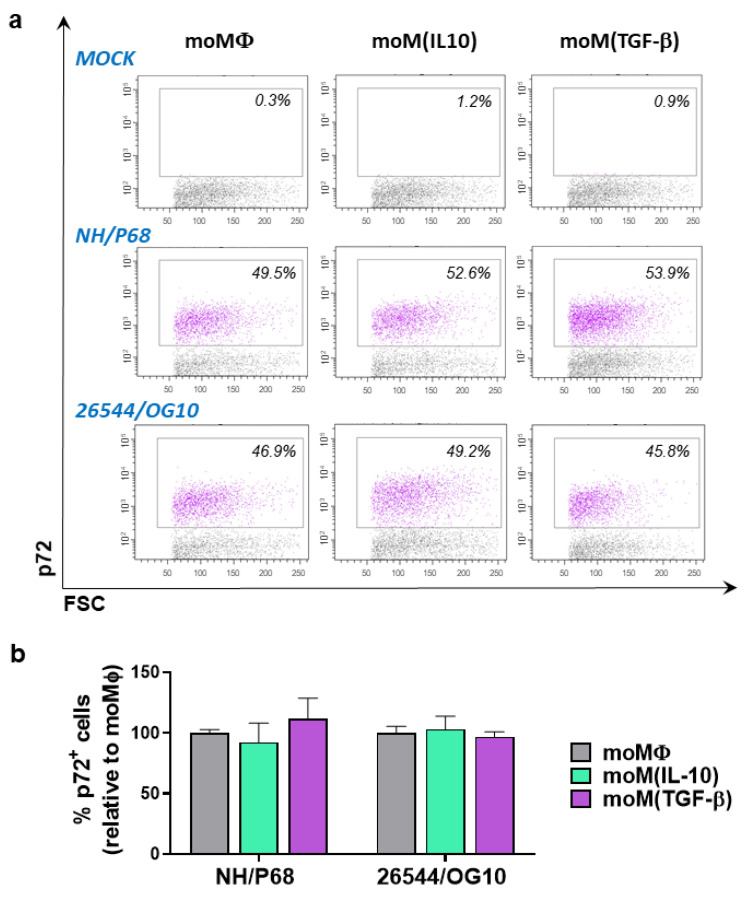
Comparison of the ability of IL-10 or TGF-β to modulate ASFV susceptibility of porcine monocyte-derived macrophage subsets. moMΦ, moM(IL-10), and moM(TGF-β) were mock infected or infected with the low virulence NH/P68 or the virulent 22544/OG10 ASFV strains, using an MOI of 1. Then, 21 hpi flow cytometry was performed to determine intracellular levels of ASFV late protein p72. Representative dot plots of mock-infected or ASFV-infected macrophages are presented in panel (**a**), whereas in panel b histograms display percentages of ASFV p72^+^ cells (**b**). In panel a, p72^+^ cell are displayed in purple, whereas p72^-^ cell are displayed in grey. In panel b, the mean data + SD from four independent experiments utilizing different blood donor pigs are presented. For each isolate (NH/P68 or 26544/OG10), values of treated macrophages were compared to the corresponding untreated control (moMΦ) using a one-way ANOVA followed by Dunnett’s multiple comparison test.

To further assess whether ASFV infected macrophage subsets with different efficiencies, we employed flow cytometry to quantify both the proportion of cells containing the ASFV late protein p72 and the relative amount of p72 (mean fluorescence intensity (MFI) of p72^+^ cells). Macrophage subsets were infected with either attenuated NH/P68 or virulent 26544/OG10 using an MOI of 1 and analyzed at 21 hpi. As displayed in Figure 3 and Figure S2, the tested immunosuppressive cytokines did not alter either the percentages or MFI of p72^+^ cells for either ASFV NH/P68 or 26544/OG10, confirming the null modulation of IL-10 or TGF-β on moMΦ susceptibility to infection at an MOI of 1.

### 2.3. Impact of IL-10 and TGF-β on moMΦ Responses to ASFV Strains of Different Virulence

With the purpose of further characterizing the impact of these immunosuppressive cytokines on macrophage responses to ASFV strains of diverse virulence, ASFV’s modulation of macrophage viability, surface marker expression, and ability to release cytokines and chemokines were investigated. Macrophage subsets were infected with NH/P68 or 26544/OG10 using an MOI of 1; mock-infected cells were used as controls. 

At 21 hpi, viability was investigated using a non-radioactive test. High percentages of live macrophages were observed in both infected and uninfected moMΦ (Figure S3). We observed that pretreatment with IL-10 or TGF-β did not influence the impact of ASFV infection on moMΦ viability (Figure S3).

Then, 21 hpi flow cytometry was employed to determine surface marker expression (alongside the intracellular levels of late viral protein p72), as was carried out as in our previous study on moMΦ [8]. At 21 hpi, cytokine and chemokine levels in culture supernatants were also evaluated by ELISA. Both cytokines downregulated MHC II and CD14, in accordance with our previous work [11], and the effect was still appreciable in the mock-infected control 21 h after the removal of the stimuli (IL-10 or TGF-β) (Figure S4). IL-10, but not TGF-β, was able to upregulate CD16 and CD163, as we previously observed [11], although differences between subsets were only statistically significant for CD16 in the mock-infected control (21 h after removal of the media supplemented with IL-10 or TGF-β) (Figure S4).

**Figure 4:**
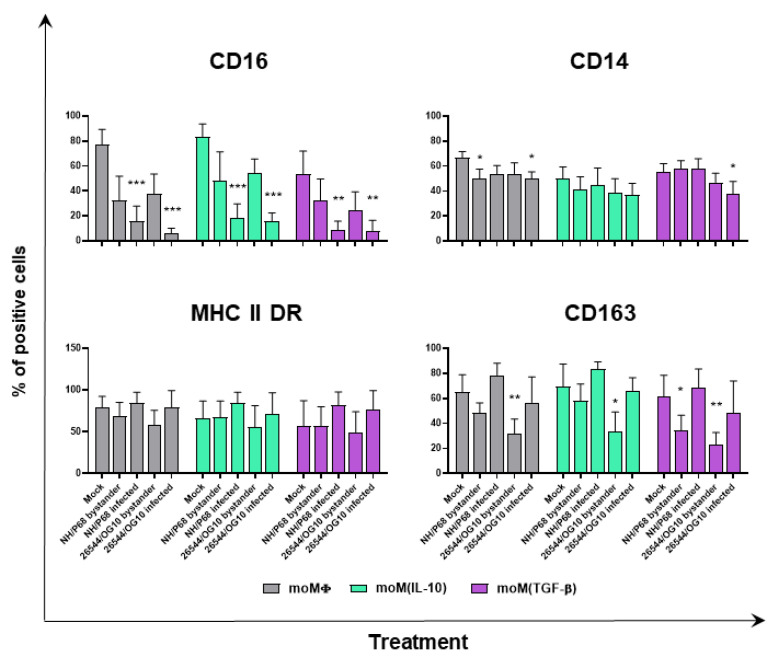
Effect of ASFV on surface marker expression (percentage of positive cells) on different macrophage subsets. moMΦ, moM(IL-10), moM(TGF-β) were mock infected or infected with the low virulence NH/P68 or the virulent 22544/OG10 ASFV isolates, using an MOI of 1. At 21 hpi, surface expression of CD16, CD14, CD163, and MHC II DR, alongside intracellular levels of ASFV late protein p72, were assessed by flow cytometry. The mean data + SD from three independent experiments utilizing different blood-donors are shown. For each marker, percentages of positive cells are displayed. Data were analyzed using a Kruskal–Wallis test followed by Dunn’s multiple comparison test, and values of ASFV-infected or bystander cells were compared to the corresponding mock-infected control; *** *p* < 0.001, ** *p* < 0.01, * *p* < 0.05.

**Figure 5:**
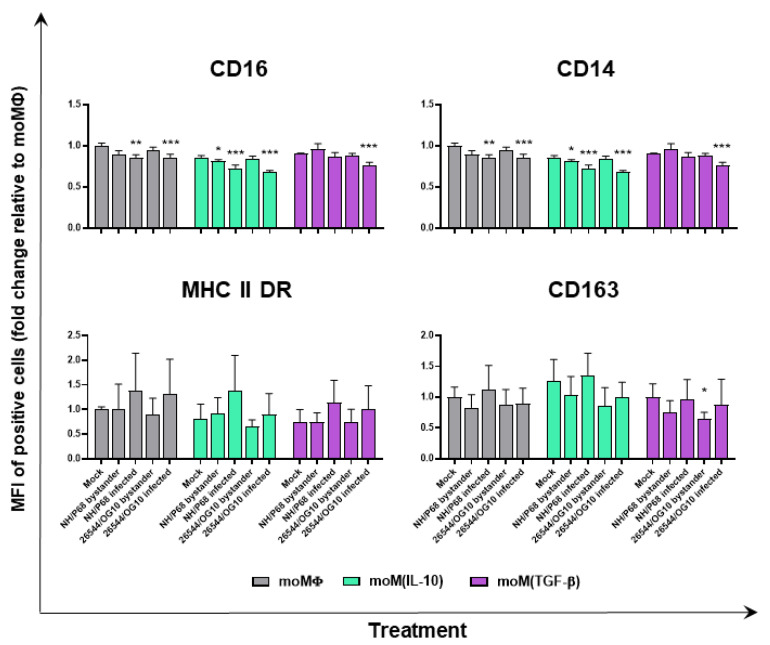
Effect of ASFV on surface marker expression (intensity of expression) on different macrophage subsets. moMΦ, moM(IL-10), moM(TGF-β) were mock infected or infected with the low virulence NH/P68 or the virulent 22544/OG10 ASFV isolates, using an MOI of 1. At 21 hpi, surface expression of CD16, CD14, CD163, and MHC II DR, alongside intracellular levels of ASFV p72 were assessed by flow cytometry. The mean data + SD from three independent experiments utilizing different blood-donors are presented. For each marker, mean fluorescence intensity (MFI) of positive cells are shown. Results are expressed as fold change relative to the mock-infected, inactivated condition (moMΦ mock). Values of ASFV-infected or bystander macrophages were then compared to the corresponding mock-infected control using a one-way ANOVA followed by Dunnett’s multiple comparison test or a Kruskal–Wallis test followed by Dunn’s multiple comparison test; *** *p* < 0.001, ** *p* < 0.01, * *p* < 0.05.

Negligible alterations in the MHC class II DR expression on macrophage subsets were detected following ASFV infection. Neither NH/P68 or 26544/OG10 modulated the expression of this marker, irrespective of macrophage activation status, but little differences were observed between infected (p72^+^) and bystander (p72^−^) moMΦ, with p72^+^ cells presenting higher levels of MHC class II DR than uninfected bystander (p72^−^) cells (Figure 4 and Figure 5). Similar results were observed for CD163, with ASFV-infected cells (p72^+^) expressing higher levels of this marker compared to uninfected bystander cells (p72^−^) (Figure 4 and Figure 5). Interestingly, infection with 26544/OG10 led to a statistically significant downregulation of CD163 on all macrophage subsets (Figure 4 and Figure 5). It was already reported that ASFV downregulated the expression of CD14 and CD16 on infected Mφ, which might result in impaired antimicrobial and antiviral activities [3,4]. In accordance with these previous reports, we observed that ASFV infection resulted in reduced expression of both markers and, in particular, infection drastically decreased CD16 levels, with marked differences in terms of the percentages of positive cells: infected (p72^+^) cells presented lower levels of CD16 compared to either mock-infected or uninfected bystander (p72^−^) cells (Figure 4 and Figure 5). Our results revealed that neither IL-10 nor TGF-β affected ASFV’s modulation of these markers (Figure 4 and Figure 5).

**Figure 6:**
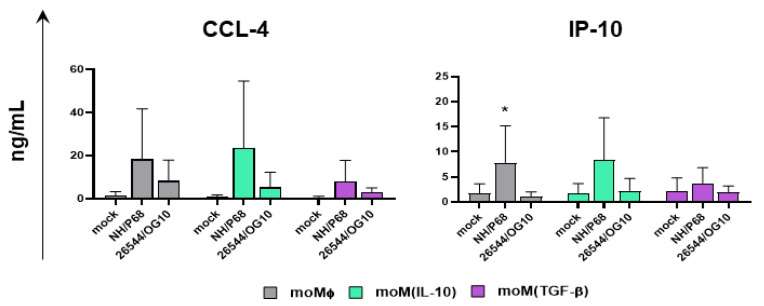
Chemokine responses of macrophage subsets in response to ASFV strains of diverse virulence. moMΦ, moM(IL-10), moM(TGF-β) were mock infected or infected with the low virulence NH/P68 or the virulent 22544/OG10 ASFV strains, using an MOI of 1. At 21 hpi, culture supernatants were collected, and levels of chemokines IP-10 and CCL-4 were determined through ELISA. The mean data + SD from three independent experiments using different animals are presented. For each macrophage subset, values of ASFV-infected macrophages were compared to the corresponding mock-infected control using a Kruskal–Wallis test followed by Dunn’s multiple comparison test; * *p* < 0.05.

Finally, the cytokine and chemokine responses of macrophage subsets to ASFV strains of different virulence infection were investigated. Cells were mock infected or infected with either NH/P68 or 26544/OG10 using an MOI of 1, and at 21 hpi, the cytokine (IL-1α, IL-1β, IL-6, IL-10, IL-12, TNF-α, and IFN-β) and chemokine (CCL-4, IP-10) levels in culture supernatants were quantified. In this study, we observed that the majority of the cytokines screened (IL-1α, IL-1β, IL-6, IL-10, IL-12, IL-18, TNF-α, and IFN-β) were not secreted in response to ASFV by moMΦ, moM(IL-10), or moM(TGF-β) (Figure S5). However, attenuated NH/P68 triggered a higher release of CCL-4 and IP-10 compared to mock-infected or 26544/OG10-infected moMΦ (Figure 6). Our data revealed that TGF-β slightly decreased the ability of moMΦ to release both CCL-4 and IP-10 in response to the attenuated NH/P6, although differences were not statistically significant.

## 3. Discussion

Macrophages (Mφ) are phagocytic cells that play a crucial role in both tissue homeostasis and the response to invading pathogens [19]. These cells are a key element of innate immunity and are characterized by remarkable plasticity [19]. Mφ can be differentially activated, resulting in their polarization into different functionally specialized subsets [5]. Each species has its own peculiarity, but previous studies have shown that, as in humans, porcine Mφ activated with IFN-γ (+/−LPS) were characterized by the upregulation of molecules involved in antigen presentation and the release of proinflammatory cytokines, whereas macrophage stimulation with IL-4 resulted in the enhanced expression of the enzyme arginase-1 [20,21,22]. We recently characterized the impact of two immunosuppressive cytokines (IL-10 and TGF-β) on porcine moMΦ, observing that both cytokines decreased the expression of two pro-inflammatory cytokines (IL-6 and TNF-α) and reduced the levels of both MHC II DR and CD14, reflecting their immunosuppressive activity. Stimulation with IL-10, but not TGF-β, led to the reduced expression of IL-1β and IL-12p40 and a stronger impairment of moMΦ responses to TLR2 or TLR4 agonists [11]. In the first part of this study, we implemented our previous characterization of the impact of both IL-10 and TGF-β on porcine moMΦ by analyzing the expression of nine key immune genes. We observed that both cytokines downregulated TLR3 expression, although IL-10 did so with a stronger intensity, and IL-10 also downregulated TLR9 expression. Both TLR3 and TLR9 are endosomal TLRs, involved in the recognition of pathogen-derived nucleic acids reviewed by [23]; thus, their downregulation might contribute to impaired responses to intracellular pathogens, including viruses. In addition, the differences observed between IL-10 and TGF-β support our previous results, as well as the concept that the former cytokine more strongly impairs the ability of macrophages to respond to external stimuli.

We then moved to the inner scope of the study: to evaluate the impact of these two cytokines on macrophage responses to ASFV. We first evaluated the impact of IL-10 and TGF-β on moMΦ susceptibility to infection with ASFV strains of different virulence. As stated above, both cytokines present immune-depressive abilities, and it was described that both IL-10 and TGF-β impaired host responses and promoted the survival and growth of intracellular pathogens [24,25]. It was also reported that viruses, such as hepatitis C, measles, and some herpes viruses, have developed strategies to enhance IL-10 expression (as reviewed by [24]). In pigs, previous studies described how recombinant porcine IL-10 was able to increase susceptibility to infection with porcine reproductive and respiratory syndrome virus (PRRSV) in either two-day-old monocytes [26], moMΦ, or monocyte-derived dendritic cells (moDC) [27]. In this study, we showed that IL-10 increased susceptibility to infection with attenuated ASFV NH/P68 at a low MOI (0.01); nevertheless, no differences were appreciated at later time points (48 or 72 hpi) or using an MOI of 1. Most interestingly, IL-10 had no impact on the ability of virulent 26544/OG10 to infect moMΦ. IL-10 stimulation is correlated with the enhanced expression of CD163, which is regarded as a putative receptor for ASFV [28]. Our results showed that the increased expression of CD163 was not associated with increased susceptibility to infection with the virulent strain, supporting the study of Popescu et al. (2017), which reported that CD163 expression was not necessary for ASFV infection [29].

Next, we assessed the impact of recombinant porcine IL-10 or TGF-β on ASFV ability to modulate moMΦ surface markers. We opted to monitor the expression of CD16, CD163, MHC II DR, and CD14, considering that, in our recent work, we observed that these markers were modulated by these immunosuppressive cytokines (both IL-10 and TGF-β downregulated MHC II DR and CD14, whereas IL-10 only upregulated CD16 and CD163 expression) [11]. As we observed in our previous work with other porcine macrophage subsets (moMΦ, moM(IL-4), and moM(IFN-α)), negligible differences in the MHC class II DR levels between mock-infected and ASFV-infected macrophage subsets were detected, but differences were observed between infected (p72^+^) and bystander (p72^−^) moMΦ, with p72^+^ cells presenting higher levels of this surface marker. Similar results were observed for CD163, and these differences are likely because ASFV preferably infects cells in a more mature status, as we previously speculated [8]. Nevertheless, despite this predilection, we observed that infection with the virulent 26544/OG10 downregulated CD163 expression on macrophages, irrespective of the activation status. These findings are similar to those reported by Nash (2020) in other myeloid cells (MoDC), where a reduction was observed in CD163 expression 24 h post-infection with virulent OURT88/1, compared to both mock and attenuated OURT88/3 infection [30]. We can hypothesize that virulent ASFV isolates either impair the synthesis of this receptor or promote its downregulation or extracellular release. CD163 expression is regulated by pro- and anti-inflammatory molecules, and its extracellular portion can be released in response to a variety of stimuli [31]. Future studies are needed to address this hypothesis and to analyze the potential consequences in vivo.

Infection with either attenuated NH/P68 or virulent 26544/OG10 resulted in the downregulation of both CD14 and CD16 expression, with a stronger intensity in infected (p72^+^) compared to bystander (p72^−^) moMΦ. CD14 downregulation was evident in all the subsets, even though the expression of this marker had already been reduced before infection by the action of IL-10 or TGF-β. Similar results were observed for CD16. IL-10 is known to trigger the upregulation of CD16 on macrophages, which is linked to increased phagocytic activity and polarization toward a ‘wound-healing’ phenotype [32]. We observed that ASFV, irrespective of the virulence, drastically reduced CD16 expression in all macrophage subsets. This might result in a decreased opsonizing activity of these cells, as we previously speculated [3,4]. Virulent ASFV isolate 26544/OG10 triggered the downregulation of two phagocytic receptors (CD16 and CD163), and these results are in accordance with those of Nash (2020), where a reduction in the phagocytic activity of moMΦ and moDC was observed following ASFV infection [30]. Future studies should investigate the mechanisms underlying CD16 reduction following ASFV infection and its potential implications in vivo.

Finally, the impact of these immunosuppressive cytokines on porcine moMΦ cytokine and chemokine responses to ASFV infection was investigated. Both IL-10 and TGF-β are powerful anti-inflammatory cytokines which reduce the inflammatory effects of other pro-inflammatory cytokines (such as TNF and IL-1β), as reviewed by [33]. In accordance with our previous results [8], we observed that ASFV infection did not trigger the release of the tested cytokines (IL-1α, IL-1β, IL-6, IL-10, IL-12, TNF-α, and IFN-β) by moMΦ. It appears that ASFV has developed strategies to silence macrophage defense responses in order to efficiently replicate in these cells; thus, the null release of pro-inflammatory cytokines and type I IFN in moM(IL-10) and moM(TGF-β) was expected. Interestingly, IL-10 release was also not detected. This is in accordance with our and others’ previous studies, where it was observed that both attenuated (NH/P68) and virulent (22653/14 genotype I, and Georgia 2007 genotype II) ASFV decreased the expression of IL-10 in macrophages [8,34]. We did not observe a release of IL-10 by moM(IL-10) or moM(TGF-β). In our recent work, we observed that neither recombinant IL-10 or TGF-β triggered the induction or release of IL-10 [11], which is regarded as hallmark of M(IL-10) or M(TGF-β) polarization in other species [35,36]. This is an interesting peculiarity of swine that merits further investigation.

In accordance with previous studies using other genotype I ASFV isolates (attenuated OURT88/3ASFV and virulent Benin 97/1 [37]), we observed that porcine macrophages release higher levels of two pro-inflammatory chemokines (IP-10 and CCL-4) in response to attenuated NH/P68, compared to virulent 26544/OG10 and mock-infected controls. Our data reveled that TGF-β slightly reduced the ability of moMΦ to release these chemokines, although without statistical significance. This observation confirms the immune-depressive ability of this cytokine. Both IP-10 and CCL-4 can attract mononuclear cells to the inflammatory site, and the latter chemokine is also known to promote the development of IFN-γ-producing Th1 lymphocytes [38]; thus, a slight reduction in their release might result in reduced immune surveillance and the subsequent impairment of the development of protective adaptive immune responses. In addition, we observed that the moMΦ of a tested subject released a higher amount of both chemokines in response to NH/P68, and similar findings were observed in our previous studies, where we noticed that moM1 of some pigs released higher levels of pro-inflammatory cytokines in response to NH/P68 compared to others [8,39]. Both host (e.g., age and gender) and environmental factors (e.g., seasonality) can influence cytokine responses [40], and future studies should address whether such factors can influence macrophage responses to ASFV.

Overall, our results revealed only a modest impact of IL-10 or TGF-β on moMΦ responses to ASFV, with a small, but statistically significant, increased ability of NH/P68 ASFV to infect moMΦ pre-treated with IL-10 at 24 hpi (using an MOI of 0.01) and with TGF-β tuning down the release by moMΦ of pro-inflammatory chemokines in response to NH/P68 ASFV infection, although without statistical significance. Both cytokines had no impact on macrophage interaction with the virulent 26544/OG10. We speculate that this is linked to the intrinsic ability of this virus to impair the defense responses of these cells. ASFV needs to use macrophages’ transcription and cellular machineries to synthesize viral proteins and efficiently replicate [41], and thus has developed mechanisms to drastically reduce the ability of macrophages to respond to invading pathogens or external stimuli [3,4].

## 4. Materials and Methods

### 4.1. Ethical Statement

Six cross-bred pigs (*Sus scrofa domesticus*), 6–18 months old, were used in this study. Animals were housed at the Experiment Station of Istituto Zooprofilattico Sperimentale (IZS) of Sardinia (‘Surigheddu’, Sassari, Italy), avoiding contact with other animals or untrained staff; animal health status was monitored routinely by trained veterinarians. Animal husbandry and handling procedures were carried out in accordance with the local ethics committee, following the Guide of Use of Laboratory Animals issued by the Italian Ministry of Health. Animal bleeding was authorized by the Ministry of Health (authorization n° 1232/2020-PR).

### 4.2. Generation of Porcine Monocyte-Derived Macrophages and Polarization

Macrophage cultures were obtained from blood leukocytes, using media supplemented with 50 ng/mL of recombinant human M-CSF (hM-CSF) (Thermo Fisher Scientific, Waltham, MA, USA), as previously described [11,42,43]. In brief, buffy coats were obtained from heparinized blood by centrifugation at 700× *g* for 30 min at 4 °C with no breaks. The buffy coat was then washed in red blood cell lysis buffer and then in PBS. The obtained leucocytes were resuspended in RPMI-1640 supplemented with 10% FBS, 100 U/mL penicillin, 100 µg/mL streptomycin (complete RPMI, cRPMI), and 50 ng/mL of recombinant hM-CSF, and plated in Petri dishes (2 × 10^7^ leukocytes/mL; 20 mL per Petri dish). After seven days at incubation at 37 °C 5% CO_2_, non-adherent cells were removed and discarded, whereas adherent cells were detached by gentle scraping with a pipette, and finally centrifuged at 200× *g* for 8 min. Cell were counted and viability was determined using a trypan blue and Countess Automated Cell Counter (Thermo Fisher Scientific). moMΦ were seeded in 12-well plates (Greiner CELLSTAR, Sigma-Aldrich, Saint Louis, MO, USA) (7–8 × 10^5^ live cells per well) or 4-well chamber slides (Nunc™ Lab-Tek™ Chamber Slide System, Thermo Fisher Scientific) (3 × 10^5^ live cells per well). After plating, cells were incubated for a further 24 h (at 37 °C 5% CO_2_) before stimulation. moMΦ were left untreated, or were treated with recombinant porcine IL-10 (20 ng/mL) or TGF-β (20 ng/mL) (both R&D Systems, Minneapolis, MN, USA) [11,42]. MoMΦ morphology was evaluated by phase contrast microscopy and May–Grunwald–Giemsa staining (see Section 4.3), whereas the absence of porcine circovirus (PCV)2, PRRSV, *Mycoplasma hyopneumoniae*, was confirmed by a qualitative real-time PCR (see Section 4.4).

### 4.3. Macrophage Subsets Morphology

MoMΦ were cultured in 4-well chamber slides (3 × 10^5^ live cells per well), stimulated with IL-10 or TGF-β or left untreated. Then, 24 h post-stimulation, cells were fixed with 4% paraformaldehyde, washed with PBS, and subsequently phase-contrast microscopy images were acquired using an inverted stereo microscope (Olympus IX 70, Segrate, Italy). The morphology of untreated and treated samples was further evaluated though May–Grunwald–Giemsa (MGG) staining: fixed cells were stained using the MGG method, and digital computer images were recorded at 20× using a light microscope coupled with a digital camera (Leica Microsystem, Welzlar, Germany) [42].

### 4.4. DNA/RNA Extraction and Detection of PCV2, PRRSV, Mycoplasma Hyopneumoniae Genome

Nucleic acids were extracted from moMΦ using a MagMax Core kit and MagMax96 extractor (Thermo Fisher) according to the manufacturer’s instructions; samples were kept at −20 °C until analyzed. The presence of PRRSV or *M. hyopneumoniae* genome was subsequently screened using commercial real-time PCR kits, LSI VetMAX™ PRRSV EU/NA and VetMAX™-Plus qPCR Master Mix (both Thermo Fisher Scientific), respectively. The presence of PCV2 was instead evaluated by qualitative real-time PCR, as we previously described [44], using forward primer 5′-TGGCCCGCAGTATTCTGATT-3′ and reverse primer 5′-CAGCTGGGACAGCAGTTGAG-3′ [45]. Samples with Ct values less than 40 were considered positive [44].

### 4.5. RT-qPCR

Changes in the mRNA expression profiles were monitored as reported in our previous studies [11,42,46]. Briefly, moMΦ were seeded in 12-well plates and left untreated or stimulated with recombinant IL-10 or recombinant TGF-β (both at 20 ng/mL); at 4 and 24 h post-treatment, culture supernatants were discarded, and moMΦ were lysed with buffer RTL (Qiagen, Hilden, Germany). Total RNA was then extracted using the RNeasy Mini Kit, treated with RNase-Free DNase Set, and finally eluted in 100 µL of ultrapure RNase-free water (all Qiagen, Milan, Italy). Then, 250 ng of purified RNA was used as a template for cDNA synthesis [13,42,46]. RT-qPCR was performed to evaluate gene expression, using the primer sets reported in Table S1. Real-time PCR amplification was performed in a CFX96™ Real-Time System after the reverse transcription step, using glyceraldehyde 3-phosphate dehydrogenase (GAPDH) as a reference gene [45]. In each sample, the relative expression of the test genes was calculated from Cq (quantification cycle) values, using the widely adopted 2^−ΔΔCq^ method [13,42,46].

### 4.6. Viruses

Two ASFV strains were used in this study: the virulent Sardinian field strain 26544/OG10 [47] (GenBank accession number KM102979; ASF Virus Archive, Virology, Animal Health, IZS of Sardinia, Sassari, Italy) and the low virulent non-hemadsorbing NH/P68 [48,49] (GenBank accession number NC044943; kindly furnished by Dr. Carmina Gallardo, EU ASF Reference Laboratory CISA-INIA, Madrid, Spain). Both ASFV isolates were propagated in vitro via the inoculation of the sub-confluent monolayers of two-day-old monocytes/macrophage cultures, using a 25 cm^2^ flask (Corning, New York, NY, USA) [8,50,51]. After incubation at 37 °C in 5% CO_2_ (over two or three days), supernatants were collected, pooled with a freeze–thawed cell lysates, and the resultant pool was clarified by centrifugation at 3000× *g* for 15 min. The pool was finally divided into aliquots and stored at −80 °C. Mock virus supernatants were prepared in an identical manner from uninfected cultures. Titers were determined by the serial dilution of virus suspensions on two-day-old monocyte/macrophage cultures (using 96-well plates) followed by hemadsorption (26544/OG10) or immunofluorescence staining (NH/P68), as before described [50,51]. In detail, immunofluorescence staining was performed 5 days pi, using a FITC-conjugated anti-ASFV polyclonal antibody (National Swine Fever Laboratory, Perugia, Italy), diluted at 1:200 ratio in PBS. Viral titers were determined using the Spearman–Kärber formula [8,50,51].

### 4.7. Infection of Macrophages

To determine the moMΦ subsets’ susceptibility to infection and their ability to respond to ASFV strains, moMΦ were seeded in 12-well plates (7–8 × 10^5^ live cells per well) and left untreated or treated with recombinant porcine IL-10 or TGF-β, as above described. Then, 24 h post-treatment, cells were infected with 26544/OG10 or NH/P68 ASFV at an MOI of 1. After 90 min incubation at 37 °C 5% CO_2_, the virus inoculum was removed, the macrophages were washed with RPMI-1640 medium, and fresh cRPMI was added to the wells (1.5 mL/well). Cells were cultured at 37 °C 5% CO_2_, and both cells and supernatants were collected at 21 hpi. Cells were stained with different monoclonal antibodies to perform flow cytometry (as described in Section 4.8), whereas supernatants were collected, clarified from cellular debris through centrifugation at 2000× *g* for 3 min, and then kept at −80 °C until the assessment of infectious virus levels by titration (see 4.6) and cytokine and chemokine levels by ELISA (described in 4.9) were performed. Then, at 21 hpi, viability was determined using a cytotoxic assay (see Section 4.10).

To determine the MoMΦ subsets’ ability to sustain ASFV growth, cells were instead infected with 26544/OG10 or NH/P68 ASFV at an MOI of 0.01. Cells were cultured at 37 °C 5% CO_2_, and supernatants were collected at several time post-infection: 0, 24, 48, and 72 hpi. Immediately after collection, supernatants were clarified from the cellular debris by centrifugation at 2000× *g* for 3 min, and then kept at −80 °C until the evaluation of infectious virus levels by titration was performed, as described above (Section 4.6).

### 4.8. Flow Cytometry

Flow cytometry was performed as previously described, with slight modifications [8,11,42,51]. In brief, moMΦ were harvested with ice-cold PBS with 10 mM EDTA (30 min incubation at 4 °C) and transferred into 5 mL round bottom tubes (Corning). Cells were initially stained with Zombie Aqua viability dye (BioLegend, San Diego, CA, USA) for 30 min at room temperature (RT) to discriminate live and dead cells. Then, moMΦ were washed with PBS supplemented with 0.1% bovine serum albumin (BSA), and were subsequently stained with the following murine monoclonal antibodies: anti-human CD14-PerCP-Cy5.5 (clone Tuk4; Miltenyi Biotec, Bergisch Gladbach, Germany), anti-pig MHC II DR (clone 2E9/13, Bio-Rad Antibodies, Kidlington, United Kingdom), CD16-PE (clone G7, Thermo Scientific Pierce, Rockford, IL, USA), and CD163-PE (clone 2A10/11, Bio-Rad Antibodies). MHC II DR expression was visualized via the subsequent staining with BV786 rat anti-mouse IgG2b (clone R12-3, BD Horizon BD Biosciences, Franklin Lakes, NJ, USA). All incubations were carried out for 15 min at 4 °C. After washing with PBS supplemented with 2% FBS, cells were fixed using Leucoperm (solution A), following the manufacturer’s instructions (Bio-Rad Antibodies). Then, moMΦ were permeabilized using Leucoperm (solution B), following the manufacturer’s instruction, and incubated with anti-p72-FITC (clone 18BG3, Ingenasa, Madrid, Spain). After 30 min of incubation in the dark at room temperature, cells were washed with PBS, resuspended in PBS supplemented with 2 mM EDTA, and finally were analyzed with a FACS Celesta (BD Biosciences). Then, 5000 live moMΦ were acquired. The analysis of the data was performed using BD FACS Diva Software (BD Biosciences), by the exclusion of doublets, gating on viable moMΦ, and then assessing the staining for surface markers. Gates for late ASFV protein p72 were set using the mock-infected controls (see Figure 3 and Figure S6), whereas surface markers were set using the corresponding unstained/isotype controls: unconjugated and IgG2b isotype control (Bio-Rad Antibodies) for MHC class II DR, mouse PE-IgG1 isotype control for CD16 and CD163 (ZX3, Thermo Scientific Pierce), and unstained control for CD14, as previously described [51]. The primary and secondary antibodies used for extracellular and intracellular staining are listed in Table S2, whereas the gating strategy is represented in Figure S6.

### 4.9. Cytokine and Chemokine Levels Determination

At 21 hpi, culture supernatants were collected, cleared by centrifugation (2000× *g* for 3 min) and stored at −80 °C until analyzed. Levels of IL-1α, IL-1β, L-6, IL-10, IL-12, and TNF-α were assessed using the Porcine Cytokine/Chemokine Magnetic Bead Panel Multiplex assay (Merck Millipore, Darmstadt, Germany) and a Bioplex MAGPIX Multiplex Reader (Bio-Rad, Hercules, CA, USA), following the manufacturers’ instructions, as previously described [11,42]. The levels of IFN-β were evaluated using a porcine IFN-β ELISA kit (MyBiosource, San Diego, CA, USA), whereas CCL-4 and IP-10 levels were assessed using a Porcine CCL-4 or IP-10 ELISA kit (Thermo Fisher Scientific, Waltham, MA, USA), according to the manufacturers’ instructions. For IFN-β, CCL-4, and IP-10, absorbance was read using an Epoch microplate reader (BioTek, Winoosky, VT, USA), as previously described [42,51].

### 4.10. Cell Viability

The impact of IL-10 or TGF-β MoMΦ viability (24 h post-stimulation), as well as the effect of ASFV on the viability of the three MoMΦ subsets (at 21 hpi), were determined using a non-radioactive cytotoxic assay [42]. Briefly, 24 h post-stimulation with IL-10 or TGF-β or 21 hpi, the Cytotox 96 Non-Radioactive Cytotoxicity Assay (Promega, Madison, WI, USA) was employed to determined lactate dehydrogenase (LDH) levels in the culture supernatants, according to the manufacturer’s instruction. A lysis solution provided by the manufacturer was used as a positive control, whereas absorbance was read at 492 nm using an Epoch microplate reader (BioTek).

### 4.11. Statistical Analysis

In vitro experiments were performed in technical duplicate (RT-qPCR, titration, flow cytometry, ELISA) or triplicate (cytotoxic assay), and repeated using at least three different blood donor animals. These data were first checked for normality using the Shapiro–Wilk test, and were then graphically and statistically analyzed with GraphPad Prism 8.01 (GraphPad Software Inc., La Jolla, CA, USA). Results were shown as mean and standard deviation (SD) and were analyzed using the parametric one-way ANOVA followed by Dunnett’s multiple comparison test, or the non-parametric Kruskal–Wallis test followed by Dunn’s multiple comparison test.

## Data Availability

The data presented in this study are available on request from the corresponding author.

## Supplementary Materials

The following supporting information can be downloaded at: https://www.mdpi.com/article/10.3390/pathogens11020166/s1. Table S1. Oligonucleotide Primer Sets for Evagreen qRT Real-Time PCR. Table S2. Antibodies used for flow cytometry. Figure S1. Impact of IL-10 or TGF-β on porcine moMΦ morphology and viability. Figure S2. Mean fluorescence intensity (median value) of ASFV p72^+^ macrophage subsets. Figure S3. IL-10 or TGF-β did not alter ASFV impact on porcine moMΦ viability. Figure S4. Effect of IL-10 or TGF-β on surface marker expression 21 h after removal of the stimuli. Figure S5. Cytokine responses of macrophage subsets in response to ASFV strains of diverse virulence. Figure S6. Gating strategy used to evaluate ASFV modulation on surface markers.
