# Supplementary material for: Analyses of the Impact of Immunosuppressive Cytokines on Porcine Macrophage Responses and Susceptibility to Infection to African Swine Fever Viruses
Source: Pathogens. 2022 Jan 27;11(2):166. doi: 10.3390/pathogens11020166 (PMC8876267; doi:10.3390/pathogens11020166)
Supplement: Supplementary file 1 [file pathogens-11-00166-s001.zip › pathogens-1495561-supplementary.pdf]

Table S1. Oligonucleotide Primer Sets for Evagreen qRT Real-Time PCR.

Gene	Sequences	Reference/Accession
NFkB/p65	F: 5′-CGAGAGGAGCACGGATACCA-3′<br>R: 5′-GCCCCGTGTAGCCATTGA-3′	[1]
IFN-β	F: 5′-AGTTGCCTGGGACTCCTCAA-3′<br>R: 5′-CCTCAGGGACCTCGAAGTTCAT-3′	[2]
TLR1	F: 5'-AGA TTT CGT GCC ACC CTA TG-3'<br>R: 5'-CCT GGG GGA TAA ACA ATG TG-3'	NM_001031775.1
TLR3	F: 5′-TGAAGAACTTGATTTCCTTGGCA-3′<br>R: 5′- GGCATGAAAACACCCTGGAG-3′	[3]
TLR5	F: 5′-TCAAAGATCCTGACCATCACA-3′<br>R: 5′-CCAGCTGTATCAGGGAGCTT-3′	[1]
TLR7	F: 5′-GTGGAAATTGCCCTCGTTGT-3′<br>R: 5′-GATGGATCTGTAGGGGAGCA-3′	[3]
TLR8	F: 5′-AAGACAACCAGTTACGTGAAATACC-3′<br>R: 5′-GGGTGTTAAAAGATAATGACAGCAC-3′	[4]
TLR9	F: 5′-AGGACTTCATGCCAAACTGC-3′<br>R: 5′-CGAGCAAACATCTCCGACTG-3′	[3]
NOS2	F:5′-CGTTATGCCACCAACAATGG-3′<br>R:5′-AGACCCGGAAGTCGTGCTT-3′	[3]
B2M	F: 5′ CGCCCCAGATTGAAATTGATTTGC-3′<br>R: 5′ GCTATACTGATCCACAGCGTTAGG-3′	[5]
GAPDH	F: 5′-ACCCAGAAGACTGTGGATGG-3′<br>R: 5′-ACGCCTGCTTCACCACCTTC-3′	[5]
HPTR1	F: 5′-TTCCTTGGTCAAGCAGCATAATCC-3′<br>R: 5′-AAGGGCATAGCCTACCACAAAC-3′	[5]

Table S2. Antibodies used for flow cytometry.

Antibody	Reactivity	Clone	Isotype	Conjugate	Concentration (mg/mL)	Working dilution*
**Primary Antibodies**						
CD14	Human	Tuk4	Mouse IgG2a	Per-CP	ND	1/5
MHC II DR	Pig	2E9/13	Mouse IgG2b	-	0.1	1/25
CD163	Pig	2A10/11	Mouse IgG1	PE	ND	1/4
CD16	Pig	G7	Mouse IgG1	PE	ND	1/4
P72	ASFV late protein P72	18BG3	Mouse IgG2a	FITC	1	1/20
**Secondary Antibodies**						
Anti-IgG2b	Mouse	R12-3	Rat IgG2a	BV786	0.2	1/20

ND: not determined. * 10 μL of diluted antibody were added to cell pellets (100 μL total).

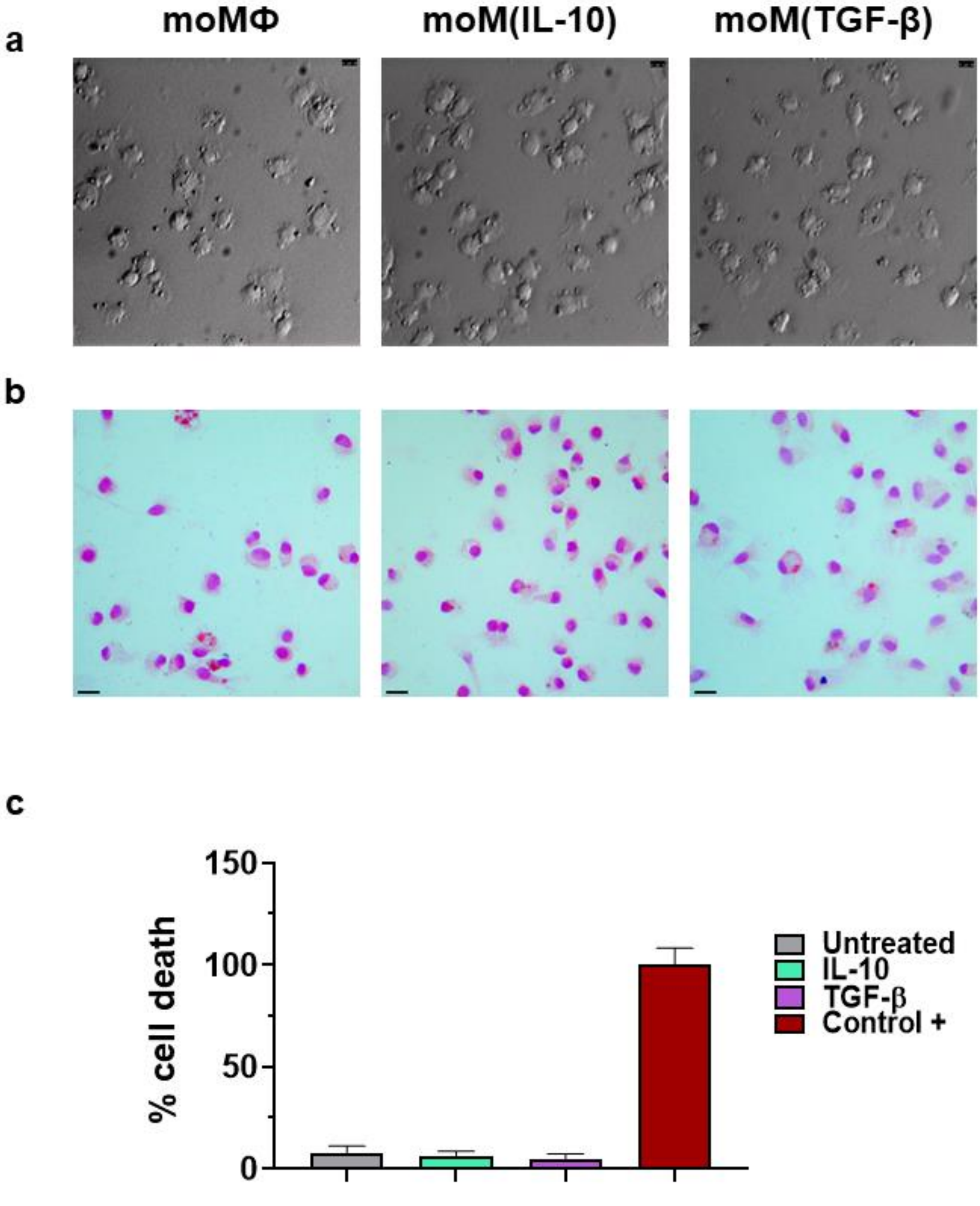

Figure S1. Impact of IL-10 or TGF-β on porcine moMΦ morphology and viability. Porcine moMΦ were left untreated or stimulated with IL-10 or TGF-β. After 24 h, morphology and viability were evaluated. (a) Phase contrast microscopy images were acquired using an inverted microscope, with a magnification 20x. Scale bar, 10 µm. (b) May-Grunwald-Giemsa staining images were acquired, with magnification 20x. Scale bar 25 µm. For both a and b, images of representative moMΦ, moM(IL-10), moM(TGF-β) are displayed. (c) MoMΦ viability was determined using a non-radioactive cytotoxic assay. A lysis solution provided by the manufacturer was used as positive control (Control +). For c, mean data + SD from three independent experiments utilizing different blood donor pigs are shown. Values of IL-10 or TGF-β-treated samples were compared to the untreated control using a Kruskal-Wallis test followed by Dunn's multiple comparison test.

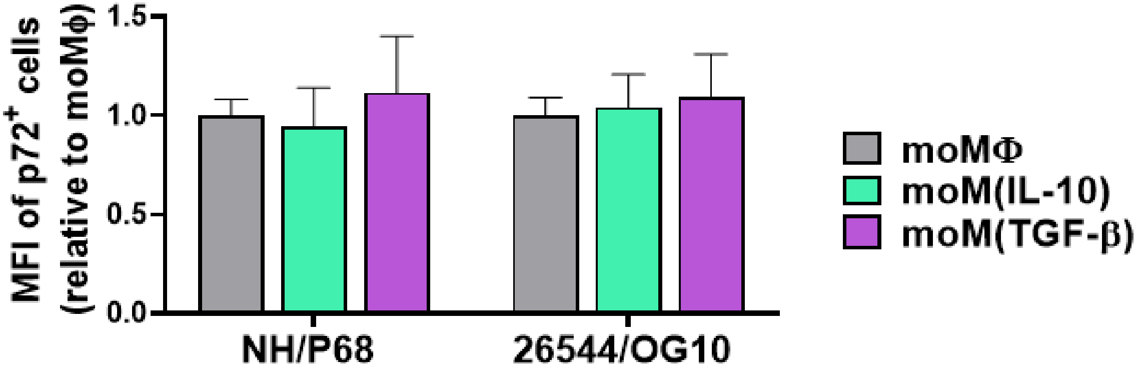

Figure S2. Mean fluorescence intensity (median value) of ASFV p72⁺ macrophage subsets. moMΦ, moM(IL-10), moM(TGF-β) were mock-infected or infected with the low virulence NH/P68 or the virulent 22544/OG10 ASFV strains, using an MOI of 1. 21 hpi flow cytometry was performed to determine intracellular levels of ASFV late protein p72 and histograms display the mean fluorescence intensity (median value) of ASFV p72⁺ cells. Mean data + SD from four independent experiments utilizing different blood donor pigs are shown. For each isolate (NH/P68 or 26544/OG10), values of treated macrophages were compared to the corresponding un-treated control (moMΦ), using a one-way ANOVA followed by Dunnett's multiple comparison test.

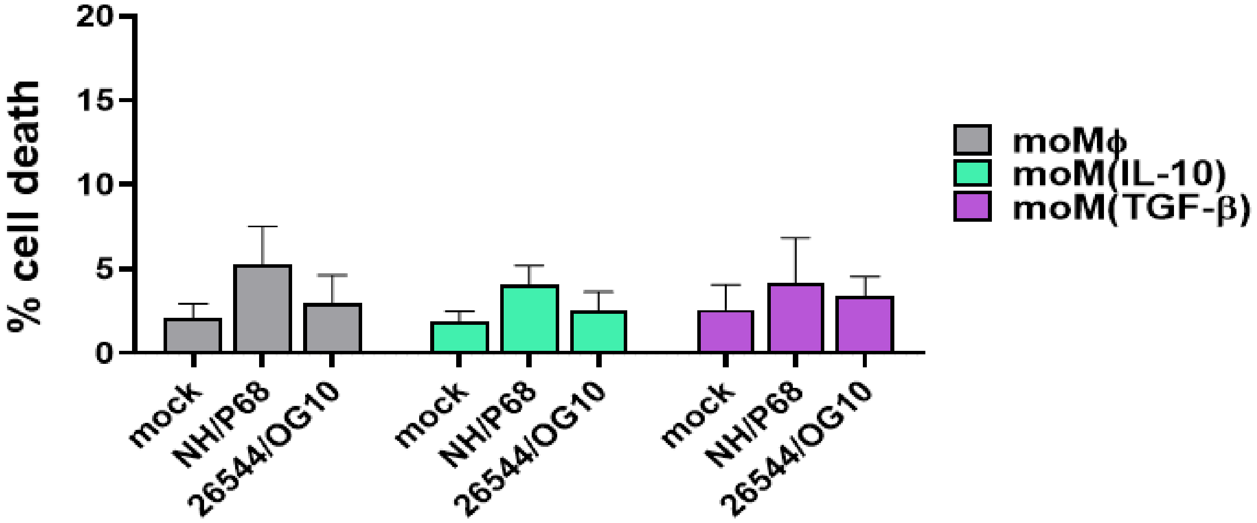

Figure S3. IL-10 or TGF-β pre-treatment does not alter the impact of ASFV on porcine moMΦ viability. moMΦ, moM(IL-10), moM(TGF-β) were mock-infected or infected with the low virulence NH/P68 or the virulent 22544/OG10 ASFV strains, using an MOI of 1. 21 hpi viability was determined using a non-radioactive cytotoxic assay. Mean data + SD from three independent experiments utilizing different blood donor pigs are shown. For each ASFV isolate (NH/P68 or 26544/OG10), values of treated macrophages were compared to the corresponding un-treated control (moMΦ), using a Kruskal-Wallis test followed by Dunn's multiple comparison test.

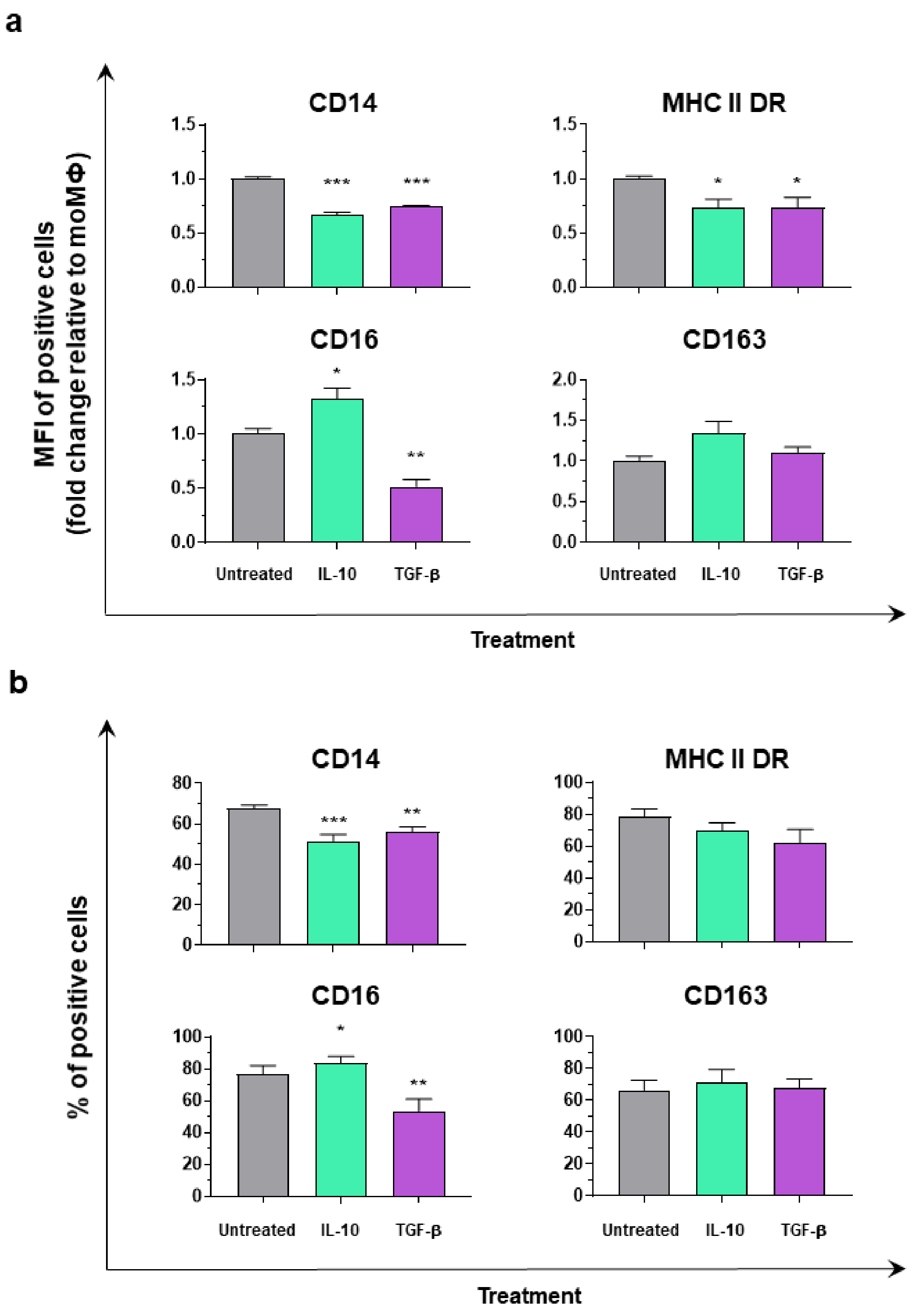

Figure S4. Effect of IL-10 or TGF-β on porcine monocyte-derived macrophage surface marker expression 21h after removal of the stimuli. moMΦ were left untreated of stimulated 24h with IL-10 or TGF-β (both at 20 ng/mL), culture supernatants were removed, and cells were mock-infected. 21 h later, surface expression of CD16, CD14, CD163, MHC II DR were assessed by flow cytometry. The mean data + SD from three independent experiments utilizing different blood-donors are shown. For each marker, mean fluorescence intensity (median value) of positive cells (a) and percentages of positive cells (b) are displayed. In panel a, values are presented as fold change relative to the mock-infected un-activated condition (moMΦ mock). Values ages were compared to the corresponding untreated control, using a using a one-way ANOVA followed by Dunnett's multiple

comparison test or a Kruskal-Wallis test followed by Dunn's multiple comparison test; *** $p < 0.001$, ** $p < 0.01$, * $p < 0.05$.

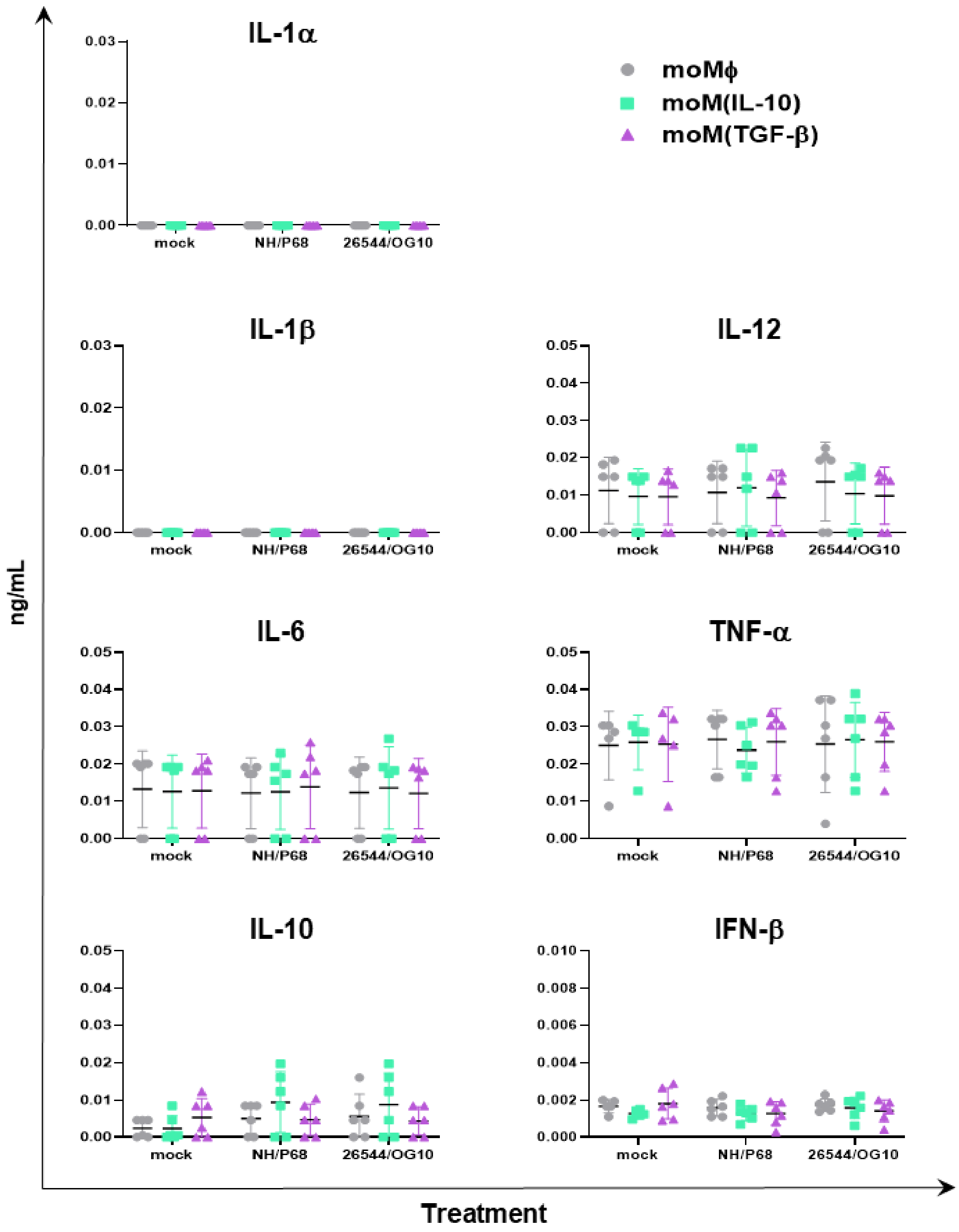

Figure S5. Cytokine responses of macrophage subsets to ASFV strains of diverse virulence. moMΦ, moM(IL-10), moM(TGF-β) were mock-infected or infected with the low virulence NH/P68 or the virulent 22544/OG10 ASFV strains, using an MOI of 1. At 21 hpi culture supernatants were collected, and levels of IL-1α, IL-1β, IL-6, IL-10, IL-12, TNF-α, IFN-β were determined using a multiplex or singleplex ELISA. The mean data ± SD from three independent experiments using different animals are presented. For each ASFV isolate (NH/P68 or 26544/OG10) values of treated macrophages (IL-10, TGF-β) were compared to the corresponding un-treated control (moMΦ), using a one-way ANOVA followed by Dunnett's multiple comparison test or a Kruskal-Wallis test followed by Dunn's multiple comparison test.

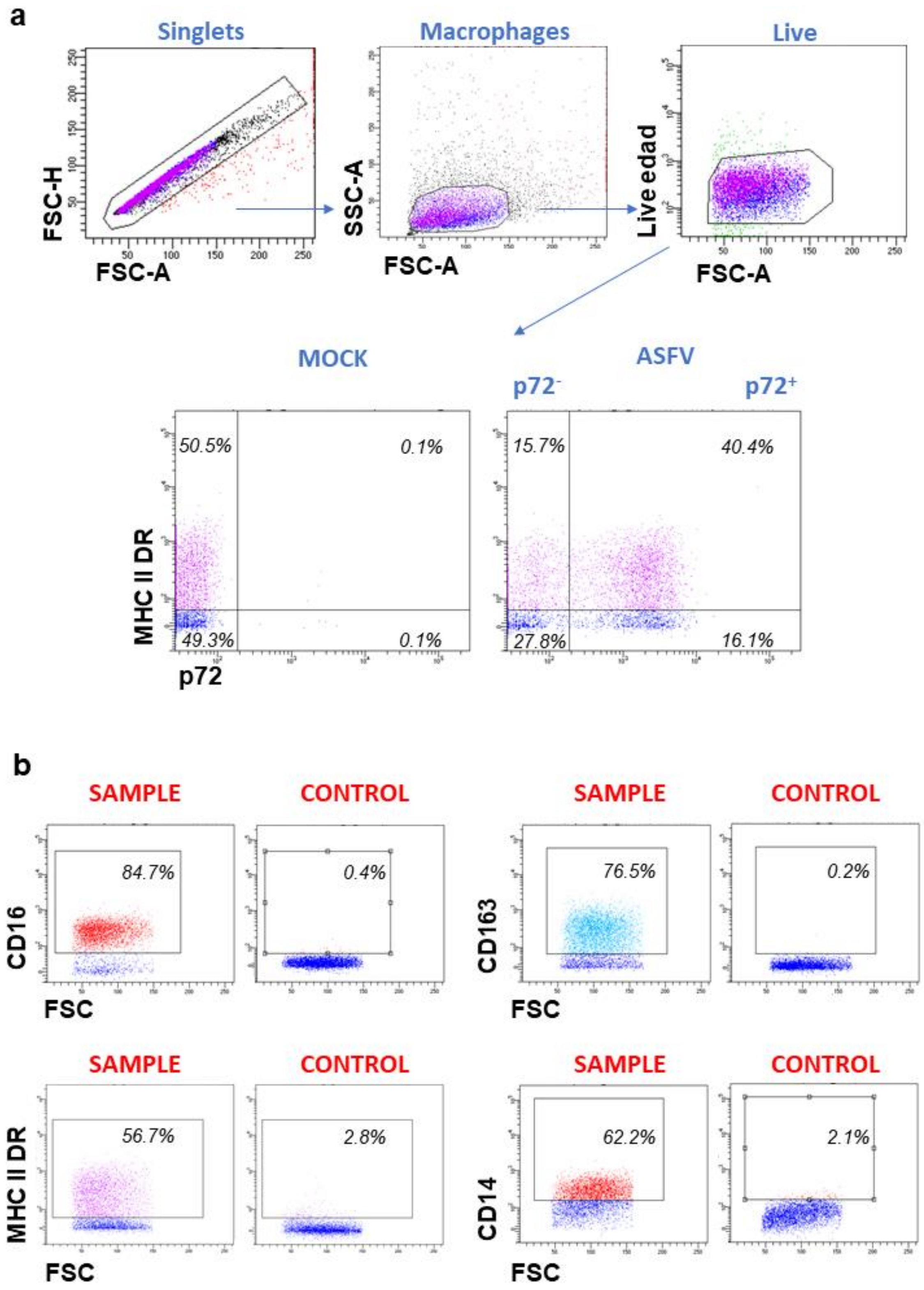

Figure S6. Gating strategy used to evaluate ASFV modulation on surface markers. In panel a gating strategy used to investigate surface marker expressions and intracellular levels of ASFV late viral protein p72 is shown. In panel b, representative dot plots of surface marker screened (CD16, CD163, MHC II DR, CD14) and corresponding controls are displayed. Gates for late ASFV protein p72 were set using the mock-infected controls (a), whereas surface markers were set using the corresponding unstained/isotype controls (b): unconjugated and IgG2b isotype control for MHC class II DR, mouse IgG1 isotype control for CD16 and CD163, and unstained control for CD14.

References

1. Razzuoli, E.; Amadori, M.; Lazzara, F.; Bilato, D.; Ferraris, M.; Vito, G.; Ferrari, A. Salmonella serovar-specific interaction with jejunal epithelial cells. *Vet. Microbiol.* **2017**, *207*, 219–225.
2. Razzuoli, E.; Villa, R.; Sossi, E.; Amadori, M. Reverse Transcription Real-Time PCR for Detection of Porcine Interferon α and β Genes. *Scand. J. Immunol.* **2011**, *74*, 412–418.
3. Franzoni, G.; Anfossi, A.; De Ciucis, C.G.; Mecocci, S.; Carta, T.; Dei Giudici, S.; Fruscione, F.; Zinellu, S.; Vito, G.; Graham, S.P.; et al. Targeting Toll-Like Receptor 2: Polarization of Porcine Macrophages by a Mycoplasma-Derived Pam2cys Lipopeptide. *Vaccines* **2021**, *9*, 692.
4. Yoo, I.; Han, J.; Lee, S.; Jung, W.; Kim, J.H.; Kim, Y.W.; Kim, H.J.; Hong, M.; Ka, H. Analysis of stage-specific expression of the toll-like receptor family in the porcine endometrium throughout the estrous cycle and pregnancy. *Theriogenology* **2019**, *125*, 173–183.
5. Razzuoli, E.; Mignone, G.; Lazzara, F.; Vencia, W.; Ferraris, M.; Masiello, L.; Vivaldi, B.; Ferrari, A.; Bozzetta, E.; Amadori, M. Impact of cadmium exposure on swine enterocytes. *Toxicol. Lett.* **2018**, *287*, 92–99.
